# Latent profiles of sleep quality, financial management behaviors, and sexual satisfaction in emerging adult newlywed couples and longitudinal connections with marital satisfaction

**DOI:** 10.3389/fpsyg.2022.883352

**Published:** 2022-08-04

**Authors:** Matthew T. Saxey, Xiaomin Li, Jocelyn S. Wikle, E. Jeffrey Hill, Ashley B. LeBaron-Black, Spencer L. James, Jessica L. Brown-Hamlett, Erin K. Holmes, Jeremy B. Yorgason

**Affiliations:** ^1^School of Family Life, Brigham Young University, Provo, UT, United States; ^2^Department of Applied Social Sciences, The Hong Kong Polytechnic University, Hong Kong, China; ^3^Oregon State University, Corvallis, OR, United States

**Keywords:** emerging adult, financial management behaviors, latent profile, marital satisfaction, newlywed, sleep quality, sexual satisfaction, financial behavior

## Abstract

Emerging adult newlywed couples often experience many demands on their time, and three common problems may surface as couples try to balance these demands—problems related to finances, sleep, and sex. We used two waves of dyadic data from 1,001 emerging adult newlywed couples to identify four dyadic latent profiles from husbands’ and wives’ financial management behaviors, sexual satisfaction, and sleep quality: *Flounderers*, *Financially Challenged Lovers*, *Drowsy Budgeters*, and *Flourishers*. We then examined how husbands’ and wives’ marital satisfaction, in relation to profile membership, varied at a later wave. We found that Financially Challenged Lovers and Flourishers had significantly higher marital satisfaction than Drowsy Budgeters and Flounderers (mostly medium effect sizes). Whereas, Financially Challenged Lovers and Flourishers did not differ in terms of marital satisfaction, Drowsy Budgeters seemed to have slightly higher marital satisfaction than Flounderers for wives only (small effect size). However, we did not find evidence that these connections meaningfully differed by sex. Implications for the efforts of clinicians and educators are discussed.

## Introduction

Although newlywed couples often begin marriage with an optimistic view of their future, couples’ experiences may not be commensurate with this view (Lavner et al., 2013). Furthermore, *emerging adult* newlywed couples (i.e., couples approximately between the ages of 18 and 29; Arnett et al., 2014) may experience unique demands on their time such as pursuing higher education, balancing career pursuits and a marriage (Ranta et al., 2014), learning how to manage finances (LeBaron and Kelley, 2021), and navigating family formation decisions (Brauner-Otto and Geist, 2018). In balancing these demands, two common relational barriers surface for these couples—problems related to money (Risch et al., 2003; Dew, 2008; Barton and Bryant, 2016) and problems related to sex (Risch et al., 2003; Rehman et al., 2011). As couples handle these two problems well, their marriage may benefit (McNulty et al., 2016; Glenn et al., 2019). Additionally, a recent report from the American Psychological Association (APA, 2021) suggests that ~67% of United States (US) adults—including emerging adults—are sleeping more or less than desired. With the added demands of emerging adulthood (e.g., Ranta et al., 2014; Brauner-Otto and Geist, 2018; LeBaron and Kelley, 2021), these newlywed couples also may sacrifice sleep to attend to other endeavors. Sleep problems matter because recent research indicates that sleep quality and marital satisfaction are positively associated (Chen et al., 2015; Lee et al., 2017; Maranges and McNulty, 2017).

Taken together, it appears that sleep quality, financial management behaviors, and sexual satisfaction each have the potential to impact emerging adult newlywed’s marital satisfaction in positive or negative ways. However, scholars have not yet examined if there are emerging adult newlywed couples that might fall into particular groups related to these constructs (e.g., proficient at money management and sleep, yet lacking in sexual satisfaction) and if group membership has implications for later marital satisfaction. To this end, we examined latent profiles of emerging adult newlywed couples’ sleep quality, financial management behaviors, and sexual satisfaction and whether profile membership is associated with later marital satisfaction to further research in this area. The remainder of the literature review is organized as follows: first, we will review theory and literature about associations between sleep quality, financial management behaviors, and sexual satisfaction predicting marital satisfaction in emerging adult newlywed couples; second, we will describe the potential for sex differences in these associations; finally, we will describe why using a person-centered approach provides unique value in examining these connections.

Scholars have found a positive association between sleep quality and marital satisfaction (Chen et al., 2015; Lee et al., 2017; Maranges and McNulty, 2017). Research with older married adults that assessed the connection between sleep quality (i.e., the degree to which one receives a healthy amount of sleep) and marital quality found a positive association between the two (Chen et al., 2015; Lee et al., 2017). In particular, scholars in one of these studies found a positive association between marital quality and sleep quality over 8 years (Lee et al., 2017), suggesting a longitudinal connection between sleep quality and marital quality. In another study, comprised of many emerging adult newlywed couples, analysis of 7 days of daily diary data suggested that on days when spouses reported getting more sleep, they also reported greater marital satisfaction (Maranges and McNulty, 2017).

Although these studies provide a foundation to build upon, further testing of sleep quality’s longitudinal impact on marital satisfaction (i.e., defined as the degree to which a spouse perceives their marriage is satisfying, rewarding, and happiness promoting) in emerging adult newlywed couples seems warranted. Although longitudinal connections between marital quality and sleep quality have been found in one study (Lee et al., 2017), a majority of studies of sleep quality and marital satisfaction have been cross-sectional (e.g., Chen et al., 2015). Because previous literature suggests the possibility of longitudinal connections between marital quality and sleep quality (Lee et al., 2017) as well as connections between sleep quality and marital satisfaction in emerging adult newlywed couples (Maranges and McNulty, 2017), we suspected that sleep quality might be longitudinally associated with marital satisfaction.

Couples and finance theory (CFT) provides insight into connections between financial management behaviors (i.e., financial behaviors that help individuals achieve financial goals and financial wellbeing; Xiao, 2016, p. 3) and marital quality (Archuleta and Burr, 2015). Indeed, one purpose of CFT is to describe the interrelated nature of financial processes—including financial management behaviors—and the couple relationship. Based on previous research that has largely shown a positive association between financial management behaviors and marital satisfaction (for a review, see Mentzer et al., 2010 and Glenn et al., 2019), CFT assumes the way a couple navigates their finances will be associated with their marital satisfaction (Archuleta and Burr, 2015). In other words, if emerging adult newlywed couples navigate potential financial problems (Risch et al., 2003; Dew, 2008; Barton and Bryant, 2016) well, their satisfaction with their relationship may benefit. Through this CFT lens, we tested if the assumption that financial management behaviors may impact marital satisfaction applies in our sample.

Previous literature supports CFT by, in general, suggesting a positive association between financial management behaviors and marital satisfaction (Mentzer et al., 2010; Glenn et al., 2019). However, much of this research has included samples of more established adults and not emerging adult newlywed couples. Indeed, qualitative evidence from couples in long-term marriages suggests that positive financial management behaviors (e.g., living within one’s means, avoiding debt, etc.) might contribute to marital quality (Skogrand et al., 2011), and quantitative evidence from adult samples supports this positive association (Spuhlera and Dew, 2019; Dew et al., 2021). One of few studies of financial management behaviors and marital satisfaction among mostly emerging adult newlywed couples also found a positive association between the two (Kerkmann et al., 2000). Furthermore, other longitudinal work suggests that when newlywed couples pay off consumer debt, their marital satisfaction likely benefits (Dew, 2008). Therefore, it is possible that financial management behaviors, which might be negatively associated with consumer debt (e.g., the better financial management behaviors are, perhaps couples might avoid consumer debt; Dew, 2008), might be longitudinally connected to marital satisfaction.

Specifically in an emerging adult context, examining financial management behaviors as playing a role, longitudinally, in impacting marital satisfaction among newlywed couples matters because achieving financial independence from family may have romantic relationship implications (Willoughby and Carroll, 2015). Although practicing healthy financial management behaviors may not be synonymous to achieving financial independence, these behaviors may help contribute toward financial independence. For example, making and sticking to a monthly budget or spending plan may help emerging adults become financially independent from their parents swifter than not doing so. Based on this previous (but limited) literature, it appeared that financial management behaviors could be longitudinally associated with marital satisfaction.

The interpersonal exchange model of sexual satisfaction (IEMSS) suggests that sexual satisfaction (i.e., defined in this study as a spouse’s satisfaction with their sexual relationship with their spouse) will likely increase when relationship satisfaction increases (Lawrance and Byers, 1995). However, recent work suggests the inverse might also have merit. That is, recent longitudinal evidence suggests that *sexual satisfaction* may also engender increases in *relationship satisfaction* (Fallis et al., 2016; McNulty et al., 2016; Cao et al., 2019a). Although the IEMSS’ assumption that romantic relationship satisfaction likely increases sexual satisfaction may also still be supported by recent research (e.g., McNulty et al., 2016), recent evidence (Fallis et al., 2016; McNulty et al., 2016; Cao et al., 2019a) seems to be suggesting the IEMSS may extend to a possible *bidirectional* association between romantic relationship satisfaction and sexual satisfaction. In this study, we tested whether the inverse of one of the main assumptions of IEMSS may apply in our sample—that is, that sexual satisfaction may be positively and longitudinally associated with marital satisfaction.

Research over the last two decades supports this possible extension by suggesting that sexual satisfaction seems to be positively associated with marital satisfaction (Fallis et al., 2016; Gadassi et al., 2016; McNulty et al., 2016; Cao et al., 2019a). Notably, Cao et al. (2019a) tested the directionality between sexual satisfaction and marital satisfaction in a sample of 268 different-sex, newlywed Chinese couples, which included many emerging adult couples. These authors found that across time, husbands’ sexual satisfaction predicted their own marital satisfaction—rather than the reverse (Cao et al., 2019a). Other studies support this longitudinal finding (i.e., sexual satisfaction predicting marital satisfaction over time) in mostly adult couples (Fallis et al., 2016) and mostly emerging adult newlywed couples (McNulty et al., 2016). Based on this previous research, it seemed reasonable to hypothesize a positive, longitudinal association between sexual satisfaction and marital satisfaction in our sample.

Additionally, it seems that for different-sex, emerging adult newlywed couples, there may be sex differences in sleep quality’s, financial management behaviors’ and sexual satisfaction’s associations with marital satisfaction. For example, Maranges and McNulty (2017) found that newlywed husbands’—but not wives’—sleep quality buffered the negative effect of daily marital evaluations (e.g., including daily evaluations of sex, chores, affection, etc.) on marital satisfaction. Put simply, high quality sleep could benefit husbands’ marital satisfaction more than high quality sleep may benefit wives’ marital satisfaction. This possible sex difference could be explained by women tending to report lower quality sleep and more disrupted sleep (Mong and Cusmano, 2016). That is, wives may be slightly more accustomed to lower quality sleep than husbands, so when husbands have lower quality sleep, it might be more likely to spill over into the marital relationship.

Furthermore, research suggests that women’s joint involvement in couple financial management longitudinally predicts relationship quality and stability (LeBaron et al., 2019). However, men’s joint involvement in financial management was not longitudinally associated with either relationship quality or stability. The authors’ feminist framework would likely support the notion that some women could have less relational power with money (i.e., women having less influence in couple financial decision making), so when women are jointly involved in the couple’s financial management, it might impact relational outcomes—like relationship quality and stability—more than when men are (LeBaron et al., 2019). Alternatively, other scholars suggest that similar sex differences (i.e., wives’ financial management behaviors as more predictive of marital outcomes than husbands’ financial management behaviors) could be due to societal expectations that husbands might be more expected to manage money well than their wives (Saxey et al., 2021). That is, because husbands might be expected to manage money well, their healthy financial management behaviors could be less predictive of marital outcomes.

Other research with different-sex, newlywed Chinese couples found that husbands’ sexual satisfaction longitudinally predicted their own marital satisfaction (Cao et al., 2019a). For wives, however, their sexual satisfaction did not longitudinally predict their own marital satisfaction. We suspect this sex difference might be due to sexual aspects of relationships tending to be more salient for men than women (Sprecher, 2002). That is, men tend to experience more pleasure (i.e., orgasm) from sex (Mahar et al., 2020), which could partially explain why sexual aspects of relationships might be more salient for men. Together, this previous literature points to the potential for sex differences in different-sex, emerging adult newlywed couples’ sleep quality’s, financial management behaviors’ and sexual satisfaction’s associations with marital satisfaction.

In this study, we did not use a traditional variable-centered approach in which researchers conduct multiple regression or structural equation modeling to examine how sleep quality, financial management behaviors, and sexual satisfaction are each associated with marital satisfaction. Instead, we used a person-centered approach to explore latent profiles of these three sets of constructs and how these latent profiles relate to marital satisfaction for the following reasons.

Methodologically speaking, a person-centered approach treats individuals, couples, and families as an undivided totality (Magnusson, 2003). That is, individuals, couples, and families are formed by the complex interactions among all key variables of the focal phenomena, and characteristics of the totality are based on the meaning of the combination of all variables of interest (Magnusson, 2003; Bergman and Trost, 2006). Specific to our study, sleep quality, sexual satisfaction, and financial management behaviors are usually intertwined. For example, recent research suggests that for newlywed husbands and wives, financial management behaviors were associated with their own sexual satisfaction (Saxey et al., 2021). Likewise, other scholars found that sleep quality was associated with financial management behaviors (O’Neill et al., 2019). Put simply, each of these three constructs can present challenges for emerging adult newlywed couples (Risch et al., 2003; Dew, 2008; Barton and Bryant, 2016; Maranges and McNulty, 2017; APA, 2021), but research has not established, for example, if there are certain couples who might navigate only two of these three challenges well, one of these challenges well, or none of these challenges well and if membership in these groups might have implications for the couple’s marital satisfaction. Thus, instead of regarding sleep quality, sexual satisfaction, and financial management behaviors as isolated constructs, it is necessary to simultaneously consider them all to address this gap in the literature.

A person-centered analysis assumes the sample is inherently heterogeneous (Bergman and Trost, 2006; Laursen and Hoff, 2006). Instead of focusing on the linear associations from each predictor to the outcome, the person-centered analysis suggests that the complex, high-order interactions among all key components shape the outcomes (Laursen and Hoff, 2006). In line with this perspective, our sample should be further classified into subgroups who share specific characteristics of the combination of critical variables of interest. The complex interactions are then interpreted as the difference in outcomes between groups that were characterized by different combinations of key variables (Bergman and Trost, 2006).

To this end, the combinations of husbands’ and wives’ sleep quality, financial management behaviors, and sexual satisfaction should be associated with marital satisfaction in a way that cannot be explained by any single variable. Although some researchers argue that the six-order interaction can be analyzed *via* a traditional moderating model (i.e., a variable-centered analysis), to unpack the complex connections between sleep quality, financial management behaviors, sexual satisfaction, and marital satisfaction, a notable advantage of a person-centered analysis is the identification of prototypical subgroups within a given sample and the ability to capture the diversity and nuances within the sample (Bauer and Shanahan, 2007).

Utilizing longitudinal, emerging adult newlywed couples data (*N* = 1,001 couples), we employed a person-centered approach to capture this nuance. That is, we explored latent profiles of husbands’ and wives’ sleep quality, financial management behaviors, and sexual satisfaction. We also examined if these latent profiles differ from each other in terms of marital satisfaction, which may provide useful evidence for educators and clinicians who help emerging adult newlywed couples, and if there might be sex differences in these connections. Based on the previously outlined theory and literature, we formulated the following research questions:

**RQ1**: Are there latent profiles of husbands’ and wives’ sleep quality, financial management behaviors, and sexual satisfaction?

**RQ2**: Is latent profile membership for husbands and wives associated with their own marital satisfaction at a later wave in statistically different ways? Said another way, across different profiles, does husbands’ and wives’ marital satisfaction vary?

**RQ3**: Are there sex differences in marital satisfaction across the latent profiles?

## Materials and methods

### Data and sample

This study used data from wave two (W2) and wave three (W3) from the Couple Relationships and Transition Experiences (CREATE) longitudinal online survey (James et al., 2021). The study’s eligibility criteria required that at least one partner in the dyad was between the ages of 18–36, this was a first marriage for at least one partner in the dyad, and that the couple lived in the US. The study collected measures from both members of the couple.

The original sample of the CREATE study at Wave 1 included 2,181 couples (90% were married in 2014, 6% were married in 2015, and 4% were married in 2013). We dropped 83 same-sex couples due to this study’s focus on different-sex couples. Additionally, because the study was focused on husbands’ *and* wives’ outcomes, 68 individuals who were not still married by W3 were dropped. Because the focus of this study was on both members of the dyad being emerging adults, 759 couples were dropped because at least one person in the couple was older than age 30 at the time of marriage. Finally, 270 couples who did not respond to the survey during W3 were dropped—leaving a final analytical sample of 1,001 couples. Table 1 provides descriptive statistics by sex for the 1,001 couples in our analytical sample. When comparing demographic characteristics of those who did and did not respond to the survey at W3, we found a few systematic differences. Responding couples were more likely to have a bachelor’s degree, more likely to have a religious affiliation, and more likely to be parents. The full results of these tests can be seen in the Supplementary Document 1.

**Table 1:** Descriptive statistics and demographic variables split by sex (*N* = 1,001 couples).

Variables	Wives			Husbands		
	Mean	SD	Min–Max	Mean	SD	Min–Max
Hispanic	0.20	0.40	0–1	0.16	0.37	0–1
White non-Hispanic	0.69	0.46	0–1	0.64	0.48	0–1
Black non-Hispanic	0.07	0.26	0–1	0.09	0.28	0–1
Other Racial/Ethnic Identity	0.04	0.26	0–1	0.11	0.29	0–1
Some College	0.76	0.43	0–1	0.67	0.47	0–1
Bachelor’s Degree	0.42	0.49	0–1	0.32	0.47	0–1
Has a Religious Affiliation	0.72	0.45	0–1	0.64	0.48	0–1
Age When Married	24.39	3.16	15–30	–	–	–
Has Children (W2)	0.55	0.50	0–1	–	–	–
Number of Children Ages 0–2	0.50	0.62	0–3			
Number of Children Ages 3–5	0.14	0.39	0–2			
Number of Children Ages 6–12	0.17	0.52	0–4			
Number of Children Ages 13–18	0.02	0.15	0–2			
Has Children with Another Partner	0.10	0.30	0–1	0.11	0.31	0–1
Credit Score (W2)	2.93	1.08	1–4	3.02	1.07	1–4
Sleep Quality (W2)	15.16	3.37	3–21	15.77	3.19	0–21
Financial Management Behaviors (W2)	3.48	0.87	1–5	3.46	0.91	1–5
Sexual Satisfaction (W2)	3.64	0.90	1–5	3.64	0.85	1–5
Marital Satisfaction (W2)	3.88	1.06	0–5	3.83	1.01	0–5
Marital Satisfaction (W3)	3.78	1.09	0–5	3.78	0.98	0–5

### Measures

#### Sleep quality

This study relied on items from the Pittsburgh Sleep Quality Index (PSQI) to measure sleep quality at W2 (Buysse et al., 1989). This scale included 19 items for respondents to rate seven aspects of sleep including: sleep quality, duration, latency, and efficiency; sleep disturbances; use of sleep medications; and daytime dysfunction. Each of the seven subscales received a score between 0 and 3. The subscales were weighted equally and summed to yield a global PSQI score between 0 and 21. For this study, we reversed the final PSQI score so that a higher score indicated better sleep quality. The PSQI is a well-known scale with a highly validated global score. Indeed, the index has good psychometric properties (Buysse et al., 1989) and has been test–retest validated (Backhaus et al., 2002).

#### Financial management behaviors

Financial management behaviors were measured at W2 using a scale with seven items from the Financial Management Behavior Scale (FMBS; Dew and Xiao, 2011), which includes approximately half of items from the original FMBS scale. The FMBS seeks to capture the frequency that participants practice productive financial behaviors, which this scale is likely to represent given its psychometric validation with nationally representative data (see Dew and Xiao, 2011). Respondents were asked how often they followed particular financial practices over the last 6 months such as whether a participant “paid all your bills on time,” “kept a written or electronic record of your monthly expenses,” or “paid off credit card balance in full each month.” Respondents rated items on a Likert scale ranging from 1 (never) to 5 (always). Higher scores indicate more robust financial management behaviors. Cronbach’s alphas suggest adequate consistency on these measures (wives: *α* = 0.75; husbands: *α* = 0.78).

#### Sexual satisfaction

The sexual satisfaction of respondents was assessed during W2 using a scale with five items. These items probed the quality of sexual intimacy within a marriage and satisfaction with the sexual relationship—although, we note that the scale was not validated. The survey asked the following five questions: “How satisfied are you with how often you currently have sex with your partner?,” “How satisfied are you with the amount of love and affection there is in your sexual relationship with your partner?,” “How satisfied are you with how often you are orgasmic during sex with your partner?,” “How satisfied are you with the amount of creativity and variety in your sexual relationship with your partner?,” and “How satisfied are you with the pattern of who initiates sex in your relationship?” Respondents rated items on a Likert scale ranging from 1 (very dissatisfied) to 5 (very satisfied). Higher scores indicate greater sexual satisfaction. Cronbach’s alphas suggest good reliability on these measures (wives: *α* = 0.83; husbands: *α* = 0.83).

#### Marital satisfaction

The marital satisfaction scale used in W2 and W3 was based on the *Couples Satisfaction Index* created by Funk and Rogge (2007). The designers of the scale used item response theory and principal component analysis in the development process. The scale has good construct validity and exhibits strong convergent validity with other relationship satisfaction scales (Funk and Rogge, 2007). The measure included four questions related to respondents’ marital satisfaction. The first three questions used a Likert scale of 0 (not at all) to 5 (completely) to assess how rewarding and satisfying their marriage is. The last question asked respondents to rate overall marital happiness on a scale of 0 (extremely unhappy) to 6 (perfect), which was scaled to lie between 0 and 5. Cronbach’s alphas suggest high consistency on these measures (wives W2: *α* = 0.95; wives W3: *α* = 0.94; husbands W2: *α* = 0.93; husbands W3: *α* = 0.91).

#### Control covariates

The analysis included several control variables. We included a respondent’s credit score from W2 as a control to account for correlations between credit score and financial management behaviors. We also included a categorical variable to capture racial/ethnic background because race may play a role in marital satisfaction (e.g., see Bryant et al., 2010). Specifically, we included four race/ethnicity categories in the study: Hispanic, White non-Hispanic, Black non-Hispanic, and all other race/ethnic groups. To control for educational attainment at W2, the analysis included a binary control for whether a respondent had attended some college (but not graduated) and another for whether the respondent had earned a bachelor’s degree or more. We also included a binary control for whether the respondent affiliated with any religion during W2. Because religious affiliation could be associated with religiosity, which is associated with marital satisfaction (e.g., Rose et al., 2018), we included religious affiliation as a control variable.

The presence of biological and non-biological children in a household may also be associated with marital satisfaction. As such, the model included a binary control for whether the couple had any children (as reported by wives in W2). To further control for the influence of children, the model included four continuous variables indicating how many children the couple had within an age range (as reported by wives in W2); the age ranges were 0–2, 3–5, 6–12, and 13–18. Additionally, the model included a binary control for each respondent from W2 indicating whether the respondent had any children with another partner. We also included a control for wives’ age at marriage and husbands’ age at marriage to account for differences in the life course of couples. Finally, we included marital satisfaction from the prior wave (W2) for each partner as controls in our analytical approach, which we describe next.

### Analytical approach

**Figure 1:**
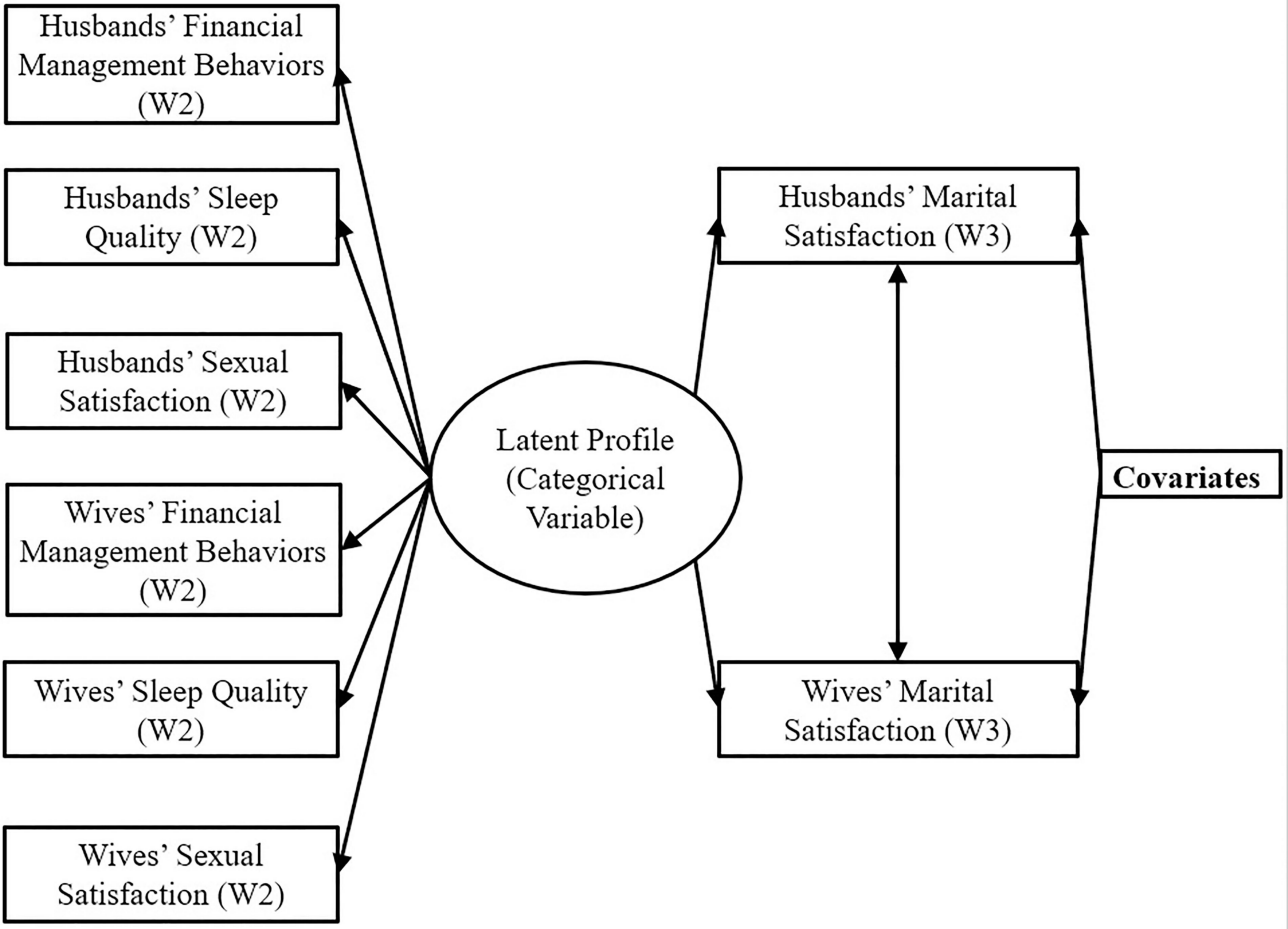
Dyadic latent profile analysis analytical model.

This study used latent profile analysis (LPA) to identify latent couple profiles (i.e., the analyses were dyadic in nature). Seen in our analytic model (Figure 1), we ran a dyadic LPA when generating latent profile memberships. That is, each couple was included as a unit and husbands’ and wives’ sleep quality, financial management behaviors, and sexual satisfaction at W2 were simultaneously included to form profiles. In this way, the identified profiles can reflect the characteristics of each couple. When estimating how latent profile membership is associated with marital satisfaction at W3, both partners’ outcomes were included in the same model, and covariance between husbands’ and wives’ marital satisfaction was estimated. Further, pathways from control variables to outcomes were estimated for both spouses. The sleep quality, financial management behaviors, sexual satisfaction, and marital satisfaction variables for husbands and wives had anywhere from 1.6% to 5.7% of missing data. Control variables also had minimal missing data, except for respondents’ credit scores, which were missing up to 25% of observations. In cases of missing data, we used Full Information Maximum Likelihood method to retain these observations.

#### RQ1: Are there latent profiles of husbands’ and wives’ sleep quality, financial management behaviors, and sexual satisfaction?

**Figure 2:**
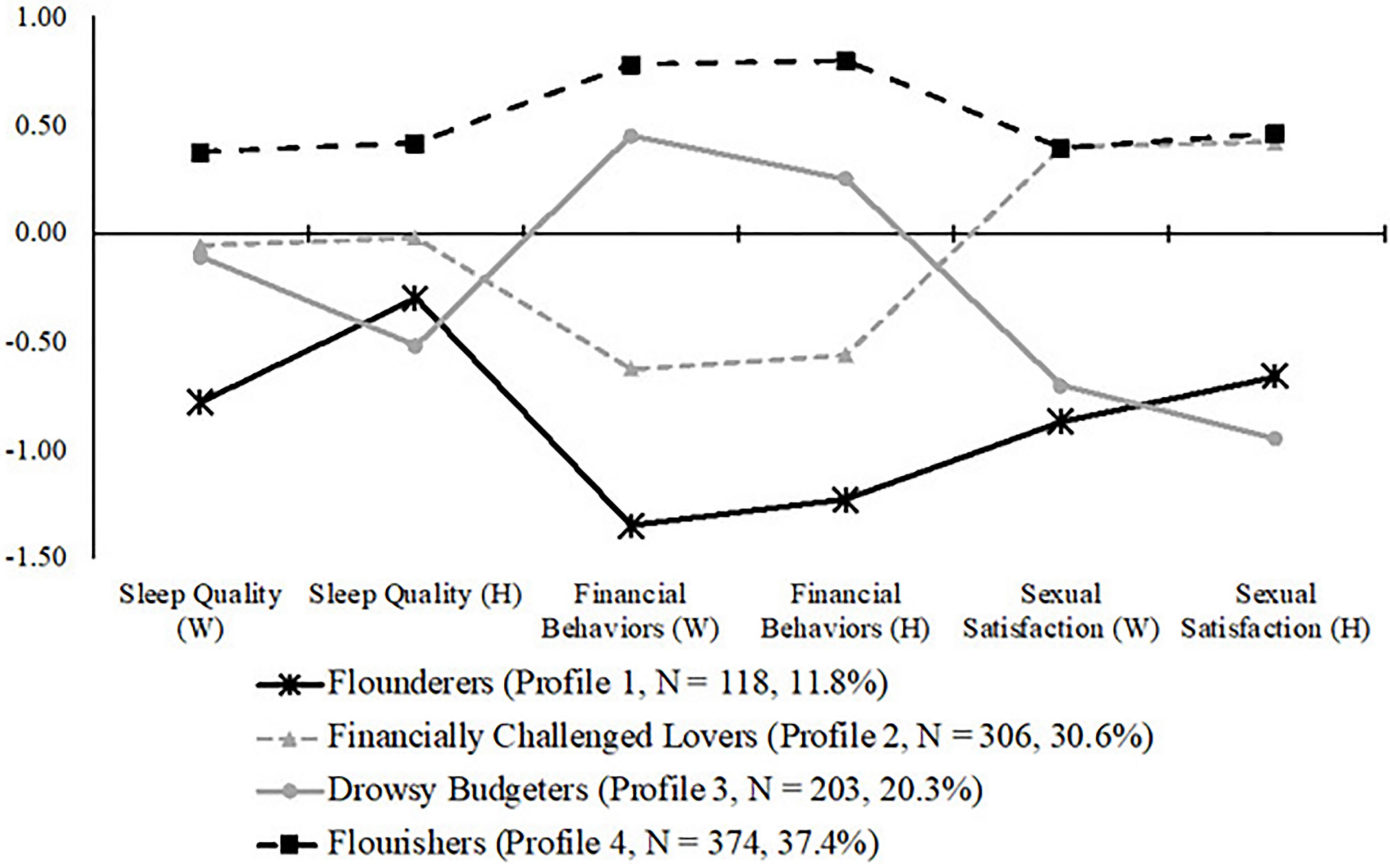
Latent profiles based on Z scores of indicators (*N* = 1,001 couples). The 0 on the *y*-axis indicates the average level across the whole sample of 1,001 couples. Positive values on the *y*-axis represent higher levels in comparison with the sample mean, and negative values on the *y*-axis represent lower levels in comparison with the sample mean. For space constraints, we labeled financial management behaviors as “financial behaviors.”

To determine the optimal number of latent profiles, we examined solutions with one to eight profiles, because models with nine or more profiles no longer converged (Weller et al., 2020). Log-Likelihood, AIC, and ABIC kept decreasing. BIC was the smallest when the number of profiles reached four, indicating the best fit (Nylund et al., 2007), and VLMRT and BLRT demonstrated that increasing the number of profiles to six or above no longer statistically significantly improved the model fit. In each model, we constrained the variance in residual of a corresponding indicator to be equal across profiles. The covariance among residuals of different indicators within each profile was fixed to zero. The four-profile model and the five-profile model were, therefore, the statistically best two models out of all eight solutions. Then, we compared the theoretical interpretability of the four-profile and the five-profile models to determine the optimal choice. Because the five-profile model did not add to common themes identified in the four-profile model, we decided to keep the four-profile model. Subsequently, we assigned profiles labels in accordance with information in Figure 2. To demonstrate the accuracy and certainty in assigning every couple to an identified profile, we calculated the average posterior class probability (AvePP > 0.70) and odds of correct classification (OCC > 5) according to statistical guidelines (Masyn, 2013) and an empirical study (Li et al., 2019).

#### RQ2: Is latent profile membership for husbands and wives associated with their own marital satisfaction at a later wave in statistically different ways?

Following the assignments to latent profiles, latent profile membership at W2 was used to assess martial satisfaction of each partner longitudinally at W3, along with other variables that may covary with profile membership. Following the recommendations in statistical papers (Asparouhov and Muthén, 2014) and empirical studies (Cao et al., 2019b), the Bolck–Croon–Hagenaars (BCH) method was used. This approach estimates differences in the husbands’ and wives’ marital satisfaction across the identified latent profile groups. In this way, all control variables mentioned in the measures section were included in the model when estimating marital satisfaction. Pairwise comparisons were conducted to detect differences in husbands’ and wives’ marital satisfaction across every latent profile (Asparouhov and Muthén, 2014). To adjust for the inflated type I error, the Bonferroni correction was utilized. Cohen’s *d*s (i.e., the effect sizes) of between-group differences were calculated using the online calculator developed by Becker (1999).

#### RQ3: Are there sex differences in marital satisfaction across the latent profiles

To detect whether the associations from latent profile membership to marital satisfaction varied between husbands and wives, we analyzed sex differences in line with prior work (Cao et al., 2019b). Specifically, and given the non-independent nature of husbands’ and wives’ reports on marital satisfaction in the same relationship, we generated a new variable—a within-couple difference score between husbands and wives—by subtracting wives’ marital satisfaction at W3 from her husbands’ marital satisfaction at W3. Then, continuing using the BCH method (Asparouhov and Muthén, 2014) and controlling for the same set of covariates, we examined whether the difference score for each identified profile differed statistically significantly from zero. Cohen’s *d*s for sex differences were calculated using the online calculator by Lenhard and Lenhard (2016) for dependent *t*-tests.

## Results

### Preliminary analyses

To examine the general associations among the main study variables, we first calculated the bivariate correlations. Seen in Table 2, statistically significant correlations were as expected. We also examined how husbands’ and wives’ financial management behaviors, sleep quality, and sexual satisfaction at W2 relate to husbands’ and wives’ marital satisfaction at W3 using an Actor-Partner Interdependence Model (APIM). Limited by scope and space, we displayed the APIM results in the Supplementary Document 2.

**Table 2:** Bivariate correlations among the main study constructs.

Construct	1	2	3	4	5	6	7
1. Husbands’ W3 marital satisfaction	–						
2. Wives’ W3 marital satisfaction	**0.62**	–					
3. Husbands’ W2 sleep quality	**0.16**	**0.13**	–				
4. Wives’ W2 sleep quality	**0.13**	**0.13**	**0.28**	–			
5. Husbands’ W2 financial management behaviors	**0.18**	**0.20**	**0.15**	**0.20**	–		
6. Wives’ W2 financial management behaviors	**0.15**	**0.23**	**0.07**	**0.24**	**0.66**	–	
7. Husbands’ W2 sexual satisfaction	**0.41**	**0.29**	**0.24**	**0.09**	**0.12**	0.04	–
8. Wives’ W2 sexual satisfaction	**0.36**	**0.40**	**0.16**	**0.18**	**0.09**	**0.12**	**0.48**

Bolded coefficients represent statistically significant (*p* < 0.05) correlations.

### RQ1: Latent profiles

Model fit information can be found in Table 3. In accordance with statistical guidelines (Spurk et al., 2020; Weller et al., 2020), we labeled each identified profile according to the standardized scores of all six indicators (three for husbands, and three for wives) for couples in each profile (see Figure 2). A positive standardized score represents high levels (within the current sample); a negative standardized score represents low levels (within the current sample); and a standardized score that is close to 0 represents average levels (within the current sample). We also estimated Wald tests to detect the within- and between- profile differences in husbands’ and wives’ reports of sleep quality, financial management behaviors, and sexual satisfaction. Given the limited space, results from the Wald tests can be seen in the Supplementary Document 3.

**Table 3:** Comparisons of models for latent profile analysis of environment coping (*N* = 1,001 couples).

Number	Log likelihood	AIC	BIC	ABIC	Entropy	VLMRT	BLRT	*N* per Profile
1	−9,975.121	19,974.242	20,033.147	19,995.035	1.000			1,001
2	−9,701.618	19,441.235	19,534.502	19,474.157	0.725	0.0000	0.0000	409, 592
3	−9,621.635	19,295.269	19,422.897	19,340.319	0.692	0.0008	0.0000	162, 358, 481
**4**	**−9,541.661**	**19,149.321**	**19,311.310**	**19,206.500**	**0.697**	**0.0005**	**0.0000**	**118, 306, 203, 374**
5	−9,518.634	19,117.267	19,313.617	19,186.575	0.721	0.0179	0000	120, 38, 192, 286, 365
6	−9,493.649	19,081.298	19,312.010	19,162.735	0.648	0.8368	0.0000	99, 115, 267, 222, 187, 111
7	−9,472.612	19,053.223	19,318.296	19,146.789	0.690	0.1206	0.0000	113, 48, 87, 265, 36, 216, 236
8	−9,452.245	19,026.490	19,325.924	19,132.185	0.682	0.1520	0.0000	83, 54, 186, 230, 135, 229, 48, 36

The bolded entries represent the fit statistics of the selected solution in the current study.

In the first profile, husbands and wives reported low levels of sleep quality, financial management behaviors, and sexual satisfaction. This profile was labeled as “*Flounderers*” (Profile 1) and was compromised of 11.8% of the sample (*N* = 118 couples; AvePP = 0.83; OCC = 29.42). In the second profile, husbands and wives reported average levels of sleep quality, low levels of financial management behaviors, yet high levels of sexual satisfaction. This profile was labeled as “*Financially Challenged Lovers*” (Profile 2) and included 30.6% of the sample (*N* = 306 couples; AvePP = 0.83, OCC = 36.20). In the third profile, husbands and wives reported high levels of financial management behaviors yet low levels of sleep quality and sexual satisfaction. This profile was labeled as “*Drowsy Budgeters*” (Profile 3) and was comprised of 20.3% of the sample (*N* = 203 couples; AvePP = 0.80, OCC = 25.13). In the fourth profile, husbands and wives reported high levels of sleep quality, financial management behaviors, and sexual satisfaction. This profile was labeled as “*Flourishers*” (Profile 4) and was comprised of 37.4% of the sample (*N* = 374 couples; AvePP = 0.86, OCC = 40.14).

### RQ2: Husbands’ and wives’ marital satisfaction across different latent profiles

Table 4 displays estimated differences in the means of marital satisfaction between each profile and the effect sizes of these differences. First, we compared all other three profiles to the Flounderers profile. In comparison with those in the Flounderers profile, wives in all other three profiles reported statistically significantly higher marital satisfaction, and effect sizes of these differences were small and medium (0.80 > Cohen’s *d*s > 0.40). Husbands in Financially Challenged Lovers and Flourishers profiles reported statistically significantly higher marital satisfaction than the Flounderers profile, and effect sizes of these differences were medium for Flourishers (Cohen’s *d* < 0.80) and large for Financially Challenged Lovers (Cohen’s *d* > 0.80). However, no statistical difference in husbands’ marital satisfaction was found between those in the Flounderers profile and those in the Drowsy Budgeters profile.

**Table 4:** Comparisons of W3 marital satisfaction between each latent profile (*N* = 1,001 couples).

	Mean	SD	Mean differences between latent profiles					
			Versus flounderers		Versus financially challenged lovers		Versus drowsy budgeters	
			Mean difference	Cohen’s *d*	Mean difference	Cohen’s *d*	Mean difference	Cohen’s *d*
Wives’ W3 marital satisfaction								
Flounderers	2.72	2.41	–	–				
Financially Challenged Lovers	4.01	1.33	1.30^***^	0.67	–	–		
Drowsy Budgeters	3.50	1.30	0.79^**^	0.41	−0.51^***^	−0.39	–	–
Flourishers	4.18	1.08	1.47^***^	0.79	0.17	0.14	0.68^***^	0.57
Husbands’ W3 marital satisfaction								
Flounderers	2.87	1.48	–	–				
Financially Challenged Lovers	4.04	1.21	1.17^***^	0.86	–	–		
Drowsy Budgeters	3.23	1.41	0.36	0.25	−0.81^***^	−0.62	–	–
Flourishers	4.24	2.31	1.37^***^	0.70	0.20	0.11	1.01^***^	0.53

Values of *p* were adjusted using the Bonferroni correction. For effect sizes, small is Cohen’s *d* from |0.20| to |0.50|, medium is Cohen’s *d* from |0.50| to |0.80|, and large is Cohen’s *d* above |0.80|.

** *p* < 0.01;

*** *p* < 0.001 (two-tailed).

Next, we compared the Drowsy Budgeters and Flourishers profile to the Financially Challenged Lovers profile. In comparison with those in the Financially Challenged Lovers profile, husbands and wives in the Drowsy Budgeters profile reported statistically significantly lower marital satisfaction, and effect sizes of these differences were small for wives (|0.50| > Cohen’s *d* > |0.20|) and medium for husbands (|0.80| > Cohen’s *d* > |0.50|). Of note, no statistical difference in husbands’ or wives’ marital satisfaction was found between those in the Flourishers profile and those in the Financially Challenged Lovers profile. Finally, we compared the Flourishers profile to the Drowsy Budgeters profile. In comparison with those in the Drowsy Budgeters profile, husbands and wives in the Flourishers profile reported statistically significantly higher marital satisfaction, and effect sizes of these differences were medium (Cohen’s *d*s > 0.50).

### RQ3: Sex differences in marital satisfaction across latent profiles

Table 5 displays the mean of difference scores between husbands’ and wives’ marital satisfaction in each profile and the Cohen’s *d*s for these difference scores. Sex differences in marital satisfaction at W3 emerged in one out of all four profiles (25%). In the Drowsy Budgeters profile, husbands reported significantly lower levels of marital satisfaction at W3 than their wives did. However, the effect size of this sex difference was very small (i.e., Cohen’s *d* = −0.16; which did not meet Cohen’s (1988) criteria of >0.20 or <−0.20 as a small-sized effect). We, therefore, concluded that the effects of profile membership on marital satisfaction were generally similar among husbands and wives.

**Table 5:** Difference scores between Husbands’ and Wives’ marital satisfaction across the latent profiles (*N* = 1,001 couples).

	Flounderers		Financially challenged lovers		Drowsy budgeters		Flourishers	
Within-couple differences	Difference	Cohen’s *d*	Difference	Cohen’s *d*	Difference	Cohen’s *d*	Difference	Cohen’s *d*
	0.13	0.06	0.02	0.02	−0.26^*^	−0.16	0.08	0.02

For effect sizes, Cohen’s *d* below |0.20| is not practically notable. Within-couple differences represent the difference between husbands’ and wives’ marital satisfaction.

* *p* < 0.05.

## Discussion

Using dyadic data from 1,001 different-sex, emerging adult newlywed couples, we explored latent profiles of husbands’ and wives’ W2 sleep quality, financial management behaviors, and sexual satisfaction and how latent profile membership was associated with their own W3 marital satisfaction. We found four latent profiles (i.e., Flounderers, Drowsy Budgeters, Financially Challenged Lovers, and Flourishers) whose martial satisfaction differed from each other in meaningful ways (i.e., in terms of effect size), which we describe next. First, we describe these four latent profiles and how these latent profiles statistically differed from each other in terms of martial satisfaction. Subsequently, we explain implications of our findings for practice.

### RQ1: Latent profile constellations

Concerning our first research question, we found four latent profiles that provide descriptive nuance to emerging adult newlywed partners’ financial management behaviors, sleep quality, and sexual satisfaction. Indeed, after accounting for relevant control variables, the following four latent profiles emerged. First, *Flounderers* (11.8% of the sample) included those husbands and wives who were low on financial management behaviors, sleep quality, and sexual satisfaction. Second, *Drowsy Budgeters* (20.3% of the sample) consisted of those husbands and wives who were low on sleep quality and sexual satisfaction yet were high on financial management behaviors. Third, *Financially Challenged Lovers* (30.6% of the sample) included those husbands and wives who reported average sleep quality, low financial management behaviors, and high sexual satisfaction. Finally, *Flourishers* (37.4% of the sample) consisted of those husbands and wives who reported high on financial management behaviors, sleep quality, and sexual satisfaction.

Our methodology and relatively large, dyadic sample allowed us to build upon previous variable-centered approaches with these constructs. Newlywed couples, especially those with unique challenges in emerging adulthood (Ranta et al., 2014; Brauner-Otto and Geist, 2018; LeBaron and Kelley, 2021), may struggle with sleep quality, financial management behaviors, and sexual satisfaction (Risch et al., 2003; Barton and Bryant, 2016; APA, 2021), which might have implications for the couple’s marital satisfaction (Dew, 2008; Maranges and McNulty, 2017; Cao et al., 2019a). However, until now, scholars have not yet examined complex interactions between these variables. Identifying these four distinct latent profiles, beyond the descriptive nuance they provided, matters because profile membership seemed to differ in terms of marital satisfaction at W3.

### RQ2 and RQ3: W2 latent profile membership, W3 marital satisfaction, and (a lack of) sex differences

In support of previous work (e.g., Dew, 2008; Lee et al., 2017; Maranges and McNulty, 2017; Cao et al., 2019a), it appears that husbands’ and wives’ sleep quality, financial management behaviors, and sexual satisfaction were collectively associated with marital satisfaction at W3, and these associations differed by profile membership—but not sex. That is, it appears that the answer to our second research question (i.e., is latent profile membership for husbands and wives associated with their own marital satisfaction at a later wave in statistically different ways?) is mostly yes, and the answer to our third research question (i.e., do these associations differ by sex?) is—rather unmistakably—no.

For wives, Financially Challenged Lovers, Drowsy Budgeters, and Flourishers each had significantly higher marital satisfaction at W3 than Flounderers (effect sizes were small and medium). For husbands, however, only Financially Challenged Lovers and Flourishers had significantly higher marital satisfaction at W3 than Flounderers (medium and large effect sizes). Overall, simultaneously having poor financial management behaviors, low sleep quality, and low sexual satisfaction seems to matter for both husbands’ and wives’ marital satisfaction at W3. When comparing Flourishers and Drowsy Budgeters to Financially Challenged Lovers, interesting differences—and a lack of difference—emerged. For both husbands and wives, Drowsy Budgeters had significantly lower marital satisfaction at W3 compared to Financially Challenged Lovers (small effect size for wives; medium effect size for husbands). For both husbands and wives, however, Financially Challenged Lovers and Flourishers did not differ from each other in terms of marital satisfaction at W3. That is, even when financial management behaviors for husbands and wives were poor, if they reported average sleep quality and high sexual satisfaction, their marital satisfaction at W3 was not statistically different from those husbands and wives high on financial management behaviors, sleep quality, and sexual satisfaction.

Although finances tend to be salient for emerging adults (LeBaron and Kelley, 2021), low versus high financial management behaviors in the Financially Challenged Lovers and Flourishers Profiles did not seem to be associated with marital satisfaction at W3 in statistically different ways. Indeed, both of these profiles were high on *sexual satisfaction*, which could be particularly salient for newlywed couples (Risch et al., 2003; Rehman et al., 2011), and this high sexual satisfaction could contribute to higher marital satisfaction even if financial management behaviors are poor. Finally, for both husbands and wives, Flourishers tended to have significantly higher marital satisfaction at W3 than Drowsy Budgeters (medium effect sizes). Although financial management behaviors were both high for these two profiles, having high sleep quality and sexual satisfaction seems to be important for marital satisfaction at W3—perhaps more important than financial management behaviors. Based on the sex difference comparisons, these connections should be considered similar across sex.

### Implications for practice

Within the context of emerging adult newlywed clients who may struggle with some combination of sleep quality, financial management behaviors, and sexual satisfaction (Risch et al., 2003; Dew, 2008; APA, 2021), we provide specific direction for clinicians who work with these clients. For example, clinicians might have use for our results as they relate to Flounderers and Drowsy Budgeters, which might include ~32% of different-sex, emerging adult newlywed couples. If an emerging adult newlywed couple is struggling with sleep quality, financial management behaviors, *and* their sexual satisfaction (i.e., Flounderers), clinicians may encourage these couples that if they prioritize improving in these three areas, their later marital satisfaction might be higher—due to the medium effect sizes of the differences between Flounderers’ and Flourishers’ marital satisfaction. However, we acknowledge that although couples’ money management and financial wellbeing have repeatedly been linked to relational wellbeing and should not be ignored (Kerkmann et al., 2000; Dew, 2008; LeBaron et al., 2019), for Flounderers, clinicians might prioritize sleep quality and sexual satisfaction in their initial sessions, given that Financially Challenged Lovers did not differ from Flourishers in their marital satisfaction.

Clinicians might also offer similar support for Drowsy Budgeters. Due to finances being a common problem for newlywed couples (Risch et al., 2003; Dew, 2008; Barton and Bryant, 2016) and emerging adults (LeBaron and Kelley, 2021), a clinician might wonder where the next evidence-based area for intervention might be for Drowsy Budgeters (i.e., which may include roughly 20% of different-sex, emerging adult newlywed couples). Our results provide specific direction for clinicians in this position to consider intervening in sleep quality and sexual satisfaction for these couples.

Indeed, sexual satisfaction appeared to be salient for marital satisfaction. That is, the two profiles with the highest marital satisfaction at W3 (i.e., Flourishers and Financially Challenged Lovers) both reported high levels of sexual satisfaction. Additionally, the supplemental APIM revealed that the only main study constructs to predict later marital satisfaction were both husbands’ and wives’ sexual satisfaction. These results further implicate the importance of helping different-sex, emerging adult clients in new marriages be intentional in their sexual relationship. To help these clients who often struggle with their sexual relationship (Risch et al., 2003), in addition to using other established therapeutic models, clinicians might consider using the sexual wholeness model (Busby et al., 2022). Using this model, clinicians might assist couples in developing a refined perspective of their sexual relationship (i.e., one that emphasizes the physical, emotional, and meaning-making aspects of couple sexuality). For each of these recommendations, along with the recommendations that follow, our results suggest that these implications are similar for husbands and wives.

Relational educators are interested in helping couples develop and sustain their romantic relationships (Stanley et al., 2020), and our findings provide descriptive nuance that may assist in these relational education efforts. For example, because sleep quality, financial management behaviors, and sexual satisfaction may be salient for emerging adult newlywed couples (Risch et al., 2003; Dew, 2008; Maranges and McNulty, 2017; APA, 2021), relational educators might be interested in teaching emerging adult newlywed couples about how to develop and sustain their romantic relationship in these areas. Describing each of the latent profiles and emphasizing how they each are longitudinally associated with marital satisfaction in different ways may help in illustrating the unique importance of sleep quality and sexual satisfaction. That is, this descriptive nuance might provide motivation to develop and sustain new marriages, especially in the areas of sleep and sex. These efforts to develop and sustain marital satisfaction at the start of new marriages may be especially important because initially high levels of marital satisfaction may remain over the first few years of marriage (Williamson and Lavner, 2020).

We note that although these latent profiles were identified net of our controls and we used longitudinal data, the constructs we examined (i.e., sleep quality, financial management behaviors, sexual satisfaction, and marital satisfaction)—and, therefore, the latent profiles—could be malleable. For example, an emerging adult newlywed couple might at one point be considered Financially Challenged Lovers or Flourishers. However, the couple may later find themselves as Flounderers or Drowsy Budgeters, which may have implications for the couple’s marital satisfaction. Just as these constructs could change in negative ways, relational educators and clinicians might also help these couples return to, or become, Flourishers. In essence, educators and clinicians who might use these findings may consider the possible malleability of these latent profiles.

### Limitations

Although this study had strengths, it also had limitations. First, since we were interested in sex differences (between wives and husbands), we only analyzed data from different-sex couples—and not the same-sex couples—in the CREATE study. Therefore, our findings may only apply to different-sex, emerging adult newlywed couples. Second, since we used a sample of emerging adult newlywed couples, our findings may not apply to more established couples, cohabiting couples, etc. Third, although we utilized longitudinal data, the associations we found among the latent profiles and marital satisfaction should not be considered causal. We also used a scale to measure sexual satisfaction that was not validated, which was a limitation of the data; future research could use more vetted measures. Finally, while the initial sample was nationally representative of newlywed couples in the US (James et al., 2021) and attrition remained minimal, attrition by W3 may have affected the sample’s representativeness.

## Conclusion

Despite the present study’s limitations, we contribute to the literature on sleep quality, financial management behaviors, and sexual satisfaction in emerging adult, different-sex new marriages. Specifically, many emerging adult, newlywed couples may fall into four particular categories in terms of financial management behaviors, sleep quality, and sexual satisfaction, which may have implications for the couple’s marital satisfaction over time. Flourishers and Financially Challenged Lovers reported the highest marital satisfaction a year later. On the other hand, Drowsy Budgeters and Flounderers reported lower marital satisfaction a year later. These findings provide novel descriptive nuance for the efforts of marital clinicians and educators. That is, as these professionals implement practices and efforts in line with these findings, the marital satisfaction of emerging adult newlywed couples may benefit.

## Data availability statement

The data analyzed in this study is subject to the following licenses/restrictions: Data from the CREATE study are not publicly available because participants have not given such consent. Questions regarding the dataset should be directed to JY (jeremy_yorgason@byu.edu).

## Ethics statement

The studies involving human participants were reviewed and approved by the Institutional Review Board at Brigham Young University. The patients/participants provided their written informed consent to participate in this study.

## Author contributions

MS wrote the Introduction and Discussion sections, edited the Method and Results sections, and collaborated with each author in completing their tasks. EJH, XL, JW, and SJ were crucial in designing the study and/or conducting the analysis—while also writing the Method and Results sections. AL-B edited and refined the Discussion. JB-H edited and refined the Introduction. EKH edited and refined the abstract. JY checked and edited the data and sample section. EKH, JY, and SJ were each crucial in collecting the data. Each of the authors (1) provided approval for publication of the content and (2) agreed to be accountable for all aspects of the work in ensuring that questions related to the accuracy or integrity of any part of the work are appropriately investigated and resolved. All authors contributed to the article and approved the submitted version.

## Conflict of interest

The authors declare that the research was conducted in the absence of any commercial or financial relationships that could be construed as a potential conflict of interest.

## Publisher’s note

All claims expressed in this article are solely those of the authors and do not necessarily represent those of their affiliated organizations, or those of the publisher, the editors and the reviewers. Any product that may be evaluated in this article, or claim that may be made by its manufacturer, is not guaranteed or endorsed by the publisher.
