# Supplementary material for: Latent profiles of sleep quality, financial management behaviors, and sexual satisfaction in emerging adult newlywed couples and longitudinal connections with marital satisfaction
Source: Front Psychol. 2022 Aug 4;13:883352. doi: 10.3389/fpsyg.2022.883352 (PMC9387670; doi:10.3389/fpsyg.2022.883352)
Supplement: Supplementary file 1 [file Table_1.DOCX]

**Supplementary Document 1**

*Demographic Comparison of Included and Excluded Couples*

|  | **Mean Included Couples (N = 1,001)** |  | **Mean Excluded Couples (N = 270)** | **Difference** |
| --- | --- | --- | --- | --- |
| W: Hispanic | 0.20 |  | 0.24 | -0.04 |
| W: White | 0.69 |  | 0.66 | **0.03*** |
| W: Black | 0.07 |  | 0.08 | -0.01 |
| W: Other Racial/Ethnic Identity | 0.04 |  | 0.02 | 0.02 |
| W: Some College | 0.76 |  | 0.78 | -0.02 |
| W: Bachelor’s Degree | 0.42 |  | 0.29 | **0.13***** |
| W: Has a Religious Affiliation | 0.72 |  | 0.58 | **0.14***** |
| W: Age When Married | 24.39 |  | 23.94 | **0.45*** |
| W: Has Children (W2) | 0.55 |  | 0.15 | **0.40***** |
| W: Has Children with Another Partner | 0.10 |  | 0.03 | **0.07***** |
| W: Credit Score (W2) | 2.93 |  | 3.67 | -0.74 |
| H: Hispanic | 0.16 |  | 0.19 | -0.03 |
| H: White | 0.64 |  | 0.73 | -0.09 |
| H: Black | 0.09 |  | 0.05 | 0.04 |
| H: Other Racial/Ethnic Identity | 0.11 |  | 0.01 | 0.10 |
| H: Some College | 0.67 |  | 0.63 | 0.04 |
| H: Bachelor’s Degree | 0.32 |  | 0.22 | **0.10**** |
| H: Has a Religious Affiliation | 0.64 |  | 0.40 | **0.24***** |
| H: Has Children with Another Partner | 0.11 |  | 0.03 | **0.08***** |
| H: Credit Score (W2) | 3.02 |  | 3.33 | -0.31 |

*Note:* *W stands for Wife, and H stands for Husband. Comparisons of differences suggested the subsample used in this study was a select group and not representative of the overall sample. * p < 0.05; ** p < 0.01; and *** p < 0.001.*
