# Supplementary material for: Latent profiles of sleep quality, financial management behaviors, and sexual satisfaction in emerging adult newlywed couples and longitudinal connections with marital satisfaction
Source: Front Psychol. 2022 Aug 4;13:883352. doi: 10.3389/fpsyg.2022.883352 (PMC9387670; doi:10.3389/fpsyg.2022.883352)
Supplement: Supplementary file 2 [file Table_2.DOCX]

**Supplementary Document 2: An Actor-Partner Interdependence Model**

We estimated an actor-partner interdependence model (APIM) to further explore the associations among variables outside of the latent groups. The same set of control variables was included, and we also estimated the autoregressive pathways from marital satisfaction at W2 to marital satisfaction at W3. As can be seen below, statistically significant results are relatively sparse and small sized, which demonstrates the necessity of using Latent Profile Analysis to examine the joint (versus separate) contributions of husbands’ and wives’ sleep quality, financial management behaviors, and sexual satisfaction to marital satisfaction.


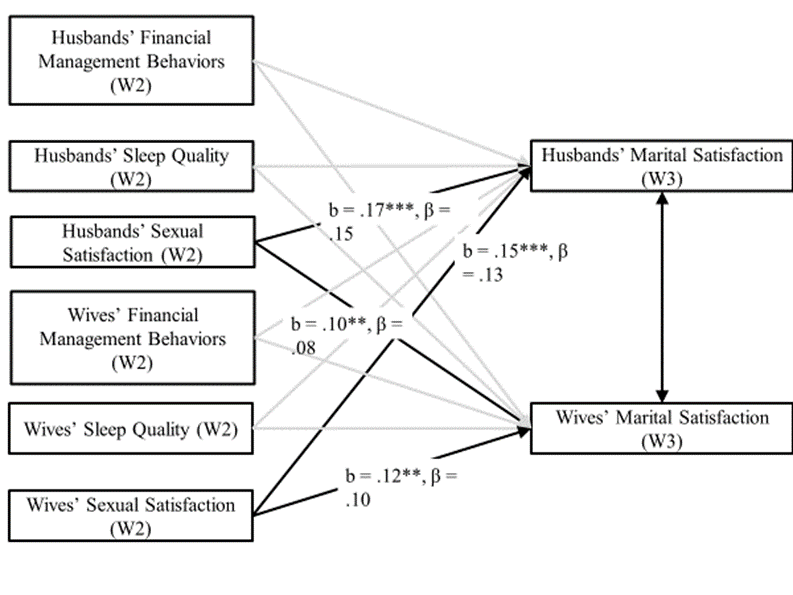


*Note: N = 1,001 different-sex newlywed couples. The APIM above fit the data relatively well: CFI = .99, RMSEA = .09, SRMR = .01. ** p < .01, and *** p < .001 (two-tailed). Grey lines represent statistically insignificant pathways.*
