# Supplementary material for: Latent profiles of sleep quality, financial management behaviors, and sexual satisfaction in emerging adult newlywed couples and longitudinal connections with marital satisfaction
Source: Front Psychol. 2022 Aug 4;13:883352. doi: 10.3389/fpsyg.2022.883352 (PMC9387670; doi:10.3389/fpsyg.2022.883352)
Supplement: Supplementary file 3 [file Data_Sheet_1.docx]

**Supplementary Document 3: Wald Tests on Husbands’ and Wives’ Reports of Sleep Quality, Financial Management Behaviors, and Sexual Satisfaction**

We estimated Wald tests as the following to detect the specific differences between and within profiles in husbands’ and wives’ reports of sleep quality, financial management behaviors, and sexual satisfaction.

For the within-profile differences between husbands and wives in sleep quality/financial management behaviors/sexual satisfaction, we only found three significant results out of 12 comparisons (25%). Such sparse findings on the sex differences within profiles demonstrated that husbands and wives were generally very similar on sleep quality/financial management behaviors/sexual satisfaction. To note, within-profile differences for the three indicators reported by husbands/wives are not independent. We used Bonferroni corrections to adjust the inflated Type I error in Wald tests.

- In Profile 1 (*Flounderers)*: Husbands reported higher sleep quality than wives.
- In Profile 3 (*Drowsy Budgeters)*: Husbands reported less responsible financial management behaviors than wives.
- In Profile 4 (*Flourishers)*: Husbands reported higher sleep quality than wives.

For the between-profile differences in the six indicators, the results are the following. The between-profile differences were relatively consistent with our labeling. For examples, partners in the “*Flourishers*” group reported highest scores on four out of six indicators; partners in the “*Financially Challenged Lovers*” group reported relatively high scores on sexual satisfaction and relatively low scores on financial management behaviors. To adjust for the inflated Type I error in Wald tests, we used Bonferroni corrections.

- Husbands’ financial management behaviors:

P4 (*Flourishers*) > P3 (*Drowsy Budgeters*) > P2 (*Financially Challenged Lovers*) > P1 (*Flounderers*)

- Husbands’ sleep quality:

No between-group differences were found.

- Husbands’ sexual satisfaction:

P4 (*Flourishers*) = P2 *(Financially Challenged Lovers*)

P4 (*Flourishers*) > P1 (*Flounderers*) > P3 (*Drowsy Budgeters*)

P2 *(Financially Challenged Lovers*) > P1 (*Flounderers*) > P3 (*Drowsy Budgeters*)

- Wives’ financial management behaviors:

P4 (*Flourishers*) > P3 (*Drowsy Budgeters*) > P2 (*Financially Challenged Lovers*) > P1 (*Flounderers*)

- Wives’ sleep quality:

No between-group differences were found.

- Wives’ sexual satisfaction:

P4 (*Flourishers*) = P2 *(Financially Challenged Lovers*)

P4 (*Flourishers*) > P3 (*Drowsy Budgeters)*

P4 (*Flourishers*) > P1 (*Flounderers*)

P2 *(Financially Challenged Lovers*) > P3 (*Drowsy Budgeters)*

P2 *(Financially Challenged Lovers*) > P1 (*Flounderers*)

P3 (*Drowsy Budgeters*) = P1 (*Flounderers*)
